# Gestational Diabetes Reduces Adenosine Transport in Human Placental Microvascular Endothelium, an Effect Reversed by Insulin

**DOI:** 10.1371/journal.pone.0040578

**Published:** 2012-07-12

**Authors:** Carlos Salomón, Francisco Westermeier, Carlos Puebla, Pablo Arroyo, Enrique Guzmán-Gutiérrez, Fabián Pardo, Andrea Leiva, Paola Casanello, Luis Sobrevia

**Affiliations:** Cellular and Molecular Physiology Laboratory (CMPL) and Perinatology Research Laboratory (PRL), Medical Research Centre (CIM), Division of Obstetrics and Gynaecology, School of Medicine, Faculty of Medicine, Pontificia Universidad Católica de Chile, Santiago, Chile; Medical University Innsbruck, Austria

## Abstract

Gestational diabetes mellitus (GDM) courses with increased fetal plasma adenosine concentration and reduced adenosine transport in placental macrovascular endothelium. Since insulin modulates human equilibrative nucleoside transporters (hENTs) expression/activity, we hypothesize that GDM will alter hENT2-mediated transport in human placental microvascular endothelium (hPMEC), and that insulin will restore GDM to a normal phenotype involving insulin receptors A (IR-A) and B (IR-B). GDM effect on hENTs expression and transport activity, and IR-A/IR-B expression and associated cell signalling cascades (p42/44 mitogen-activated protein kinases (p42/44^mapk^) and Akt) role in hPMEC primary cultures was assayed. GDM associates with elevated umbilical whole and vein, but not arteries blood adenosine, and reduced hENTs adenosine transport and expression. IR-A/IR-B mRNA expression and p42/44^mapk^/Akt ratios (‘metabolic phenotype’) were lower in GDM. Insulin reversed GDM-reduced hENT2 expression/activity, IR-A/IR-B mRNA expression and p42/44^mapk^/Akt ratios to normal pregnancies (‘mitogenic phenotype’). It is suggested that insulin effects required IR-A and IR-B expression leading to differential modulation of signalling pathways restoring GDM-metabolic to a normal-mitogenic like phenotype. Insulin could be acting as protecting factor for placental microvascular endothelial dysfunction in GDM.

## Introduction

Human placenta microvascular endothelial cells (hPMEC) maintain normal adenosine extracellular level by an efficient uptake of this nucleoside [1], thus modulating its broad biological effects [2]. hPMEC take-up adenosine via Na^+^-independent, human equilibrative nucleoside transporters 1 (hENT1, inhibited by ≤1 µmol/L nitrobenzylthioinosine, NBTI) and 2 (hENT2, inhibited by >1 µmol/L NBTI) [2]. hENT1 is down-regulated in human umbilical vein endothelial cells (HUVEC) from gestational diabetes mellitus (GDM) [3], [4]; however, adenosine transport in hPMEC from GDM has not been addressed [5]. GDM, characterized by maternal and fetal hyperglycaemia [6]–[8], associates with elevated human umbilical vein blood adenosine [4] and defective placental insulin signalling [5]–[9]. Since previous studies show increased hENT2 expression and activity in response to insulin in HUVEC from normal pregnancies [10], it is likely that insulin could modulate hENT2 in hPMEC.

Insulin activates plasma membrane insulin receptor (IR) isoforms A (IR-A) and B (IR-B) [5], [11]. These transcripts relative abundance is tissue-specific [11], suggesting that IR-A and IR-B functional differences might underlie tissue-specific insulin effect *in vivo*. IR-A is preferentially expressed in HUVEC from GDM [4], complementing similar information and increased IR-B mRNA expression in patients with diseases characterized by insulin resistance such as type 2 diabetes mellitus (T2DM) [12], [13] or myotonic dystrophy [14]. Increased mitogen-activated protein kinases 1/2 (p42/44^mapk^)/protein kinase B (Akt) ratio, i.e., higher mitogenic/metabolic-like signalling ratio [15], [16], is characteristic of higher IR-A/IR-B ratios. Since GDM is associated with umbilical vein blood hyperinsulinemia [17], [18], we hypothesize that GDM alters hENT2-mediated transport in hPMEC, and that a GDM-like phenotype will be restored to a normal-like phenotype by insulin involving IR-A or IR-B activation. Results show reduced hENT2-adenosine transport and expression, and *SLC29A2* (for hENT2) promoter activity, and reduced IR-A/IR-B expression ratio paralleled by p42/44^mapk^/Akt activation ratios in GDM, all phenomena reversed by insulin. These findings could be determinant in diseases of pregnancy associated with abnormal insulin signalling and endothelial dysfunction such as GDM [5], [7].

## Methods

### Ethics statement

The investigation conforms to the principles outlined in the Declaration of Helsinki. Ethics Committee approval from the Faculty of Medicine of the Pontificia Universidad Católica de Chile, the Comisión Nacional de Investigación en Ciencia y Tecnología (CONICYT, Chile) and patient informed written consent were obtained.

**Table 1:** Clinical characteristics of patients and newborns.

Variables	Normal (*n = *64)	GDM (*n = *64)
*Maternal variables*		
Age (years)	28±0.64 (18–38)	31±0.59 (18–38)
Height (cm)	159±1.5 (147–177)	159±0.9 (147–185)
Weight (kg)		
24–28 weeks of gestation	59 ± 3.3 (48–66)	56±2.1 (43–61)
38–40 weeks of gestation	68 ± 2.0† (54–92)	64±1.4† (45–86)
BMI (kg/m^2^)		
24–28 weeks of gestation	24±0.27 (21–27)	22±0.28 (21–26)
38–40 weeks of gestation	26±0.23† (22–29)	25±0.37† (19–29)
Systolic blood pressure (mm Hg)		
24–28 weeks of gestation	102±5 (99–106)	104±4 (99–109)
38–40 weeks of gestation	107±7 (103–110)	112±6 (105–113)
Glycosilated hemoglobin A_1c_ (% of total)		
24–28 weeks of gestation	4.2±0.32 (3.2–5.0)	4.4±0.15 (4.2–5.0)
38–40 weeks of gestation	4.0±0.34 (3.2–5.1)	5.7±0.10*† (5.3–6.2)
Glycemia basal (mg/dL)	82±0.92 (67–95)	83±1.1 (67–114)
OGTT (mg/dL)		
Glycemia basal	88±1.2 (69–97)	95±1.9 (87–112)
Glycemia 2 hours after glucose	98±1.2 (65–114)	164±2.5* (140–239)
Plasma insulin (μU/mL)	5.2±0.13 (4.9–5.6)	7.8±0.7* (5.9–11.6)
HOMA-IR	1.06±0.01 (0.8–1.3)	1.59±0.01* (1.0–3.2)
HOMA-IS (%)	94.3±0.6 (77.4–125.2)	62.9±0.4* (31.2–78.3)
ß-Cell function (%)	104±0.6 (90–120)	173±1.2* (130–215)
*Newborn variables*		
Sex (female/male)	34/30	35/29
Gestational age (weeks)	38±0.32 (37–40)	38±0.21 (37–40)
Birth weight (grams)	3192±44 (2580–3970)	4353±51* (3800–5100)
Height (cm)	49±3.6 (36–54)	49±4.1 (43–55)
Ponderal index (grams/cm^3^×100)	2.5±0.03 (2.0–3.3)	3.7±0.06* (3.1–5.7)
Umbilical vein D-glucose (mmol/L)	3.6±0.4 (2.9–4.3)	4.5±0.6 (4.1–4.9)
Umbilical vein insulin (μU/mL)	6.1±0.7 (5.5–7.9)	11.6±0.6* (8.9–13.1)
HOMA-IR	0.98±0.11 (0.70–1.50)	2.32±0.21* (1.62–2.85)
HOMA-IS (%)	102.1±11.4 (87.3–110.5)	43.1±3.9* (30.5–60.3)

Data are mean ± SEM (range), except hemoglobin A_1c_ where values are mean ± SD (range). OGTT, oral glucose tolerance test; BMI, body mass index; HOMA-IR, homeostasis model assessment for insulin resistance; HOMA-IS, homeostasis model assessment for insulin sensitivity (see Methods). *P<0.05 versus Normal. † *P*<0.05 versus values at 24–28 weeks of gestation in GDM.

### Human placentas and study groups

Placentas were collected after delivery from 64 full-term normal or 64 full-term gestational diabetic pregnancies. Patients between the 24–28 weeks of gestation with basal glycaemia <90 mg/dL (i.e., overnight starvation) and >140 mg/dL at 2 hours after an oral glucose load (75 g) were diagnosed as gestational diabetes mellitus (GDM) [4], [6]. Patients with GDM were treated with diet (1500 kcal/day and 200 g of carbohydrates as maximum per day). All pregnancies were singleton and pregnant women were normotensive, non-smoking, non-alcohol or drug consuming, and without intrauterine infection or any other medical or obstetrical complications (Table 1). Patients with GDM exhibit increased maternal glycosilated hemoglobin A_1c_, altered oral glucose tolerance test (OGTT), insulinemia, increased insulin resistance and reduced ß-cell function. Newborn from DGM exhibit increased insulin resistance and ponderal index compared with normal pregnancies.

The homeostasis model assessment for insulin resistance (HOMA-IR) [19] was calculated from:
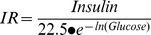
where *Insulin* is in μU/mL and *Glucose* is basal glycaemia in mmol/L [2]. Insulin sensitivity (*IS*) was derived from these values by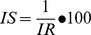 (expressed in %). Additionally, ß-cell function (*ßcf*, expressed in %) was estimated from:



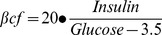



**Figure 1:**
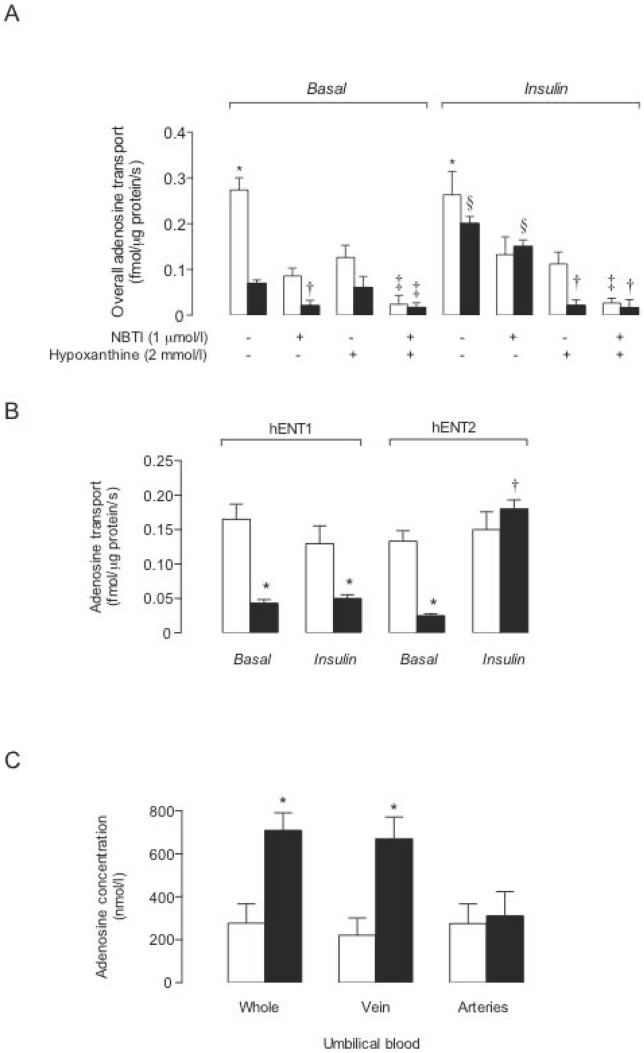
GDM and insulin effect on adenosine transport. (a) Overall (10 µmol/L) adenosine transport (hENT1+hENT2 mediated) in hPMEC from normal (white bars, Normal) or GDM (black bars) pregnancies incubated (8 hours) with basal-insulin (*Basal*) or 1 nmol/L insulin (*Insulin*) in the absence (–) or presence (+) of nitrobenzylthioinosine (NBTI) or hypoxanthine. (b) hENT1- and hENT2-mediated adenosine transport from data in (a). (c) Adenosine concentration in whole umbilical blood (Whole), umbilical vein (Vein) or arteries (Arteries) blood. In (a) (n = 16), *P<0.05 versus all other values in *Basal* or *Insulin*, except *Insulin* in the absence of NBTI or hypoxanthine. In *Basal*, †*P*<0.05 versus GDM in the absence of NBTI or hypoxanthine, and Normal in the presence of NBTI, ‡*P*<0.05 versus corresponding values in the presence of hypoxanthine. In *Insulin*, †*P*<0.05 versus GDM in the absence or presence of NBTI, or Normal in the presence of hypoxanthine. ‡*P*<0.05 versus all corresponding values in Normal. §*P*<0.05 versus all other values in GDM in *Basal*. In (b) (n = 16), *P<0.05 versus corresponding values, †*P*<0.05 versus GDM. In (c) (n = 4), *P<0.05 versus corresponding values in Normal. Values are mean ± SEM.

### Cell culture

Human placenta microvascular endothelial cells (hPMEC) were isolated as previously described [1]. In brief, confluent cells obtained from placental tissue samples (∼4 cm^3^ of the chorionic villous) digested with trypsin/EDTA (0.25/0.2%, 20 minutes, 37°C) followed by 0.1 mg/mL collagenase (2 hours, 37°C, Type II from *Clostridium histolyticum*; Boehringer, Mannheim, Germany) in medium 199 (M199, Gibco Life Technologies, Carlsbad, CA, USA), and filtered through a 55 μm pore size Nylon mesh, were trypsinized (trypsin/EDTA  = 0.25/0.2%, 3 minutes, 37°C) and subjected to CD31 (against platelet endothelial cell adhesion molecule 1, PECAM-1)-positive immunoselection using Dynabeads^©^CD31 microbeads (DYNAL, Norway). hPMEC were cultured under standard conditions (37°C, 5% CO_2_) in M199 containing 5 mmol/L D-glucose, 10% new born calf serum (NBCS), 10% fetal calf serum (FCS), 3.2 mmol/L L-glutamine and 100 U/mL penicillin-streptomycin (primary culture medium, PCM) [1]. Confluent (passage 3) cells were incubated in PCM containing 0.25% newborn and 0.25% fetal calf sera (low sera PCM) for 24 hours prior exposure (8 hours) to 1 nmol/L insulin (experimental condition hereafter referred as ‘insulin’). Since insulinemia in GDM fetal blood was higher than normal pregnancies (Table 1), in some experiments culture medium was supplemented with 0.04 nmol/L (∼5.2 µU/mL) or 0.07 nmol/L (∼7.8 µU/mL) insulin (hereafter referred as ‘basal-insulin’) for normal or GDM pregnancies, respectively. All experiments were performed in paired cell cultures from normal or GDM pregnancies. To have an estimation of a potential selection bias in our study some experiments were also performed in unpaired cell cultures from normal or GDM pregnancies. The results obtained in the latter assays were within the S.E.M. values obtained for the reported paired experiments.

**Figure 2:**
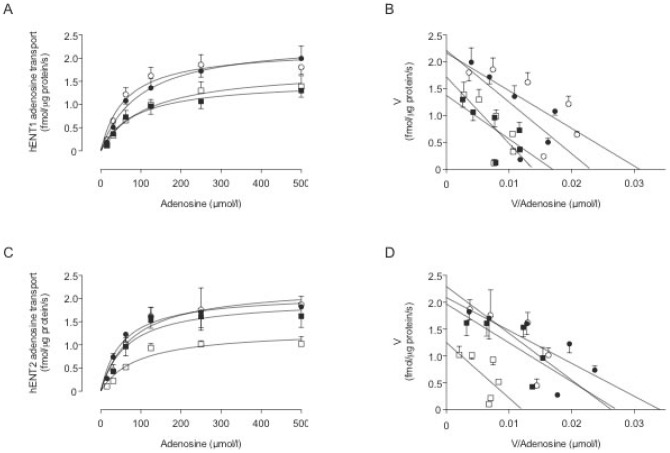
GDM and insulin effect on hENT2-mediated adenosine transport. hENT1 (a) and hENT2 (c) mediated adenosine transport in hPMEC from normal (circles) or GDM (squares) pregnancies incubated (8 hours) with basal-insulin (white symbols) or 1 nmol/L insulin (black symbols). Eadie-Hofstee plots represent hENT1 (b) or hENT2 (d) transport data in (a) and (c), respectively. Values are mean ± SEM (n = 16).

**Table 2:** Effect of GDM and insulin on adenosine transport kinetics.

Parameter	Normal			GDM	
	*Basal-insulin*	*Insulin*	*Basal-insulin*		*Insulin*
*hENT1-mediated*					
* V*_max_ (fmol/µg protein/second)	2.2±0.2	2.4±0.2	1.8±0.1*		1.5±0.1**
* K*_m_ (µmol/L)	61±16	72±18	111±20		84±18
* V*_max_ / *K*_m_ (fmol/µg protein/second/(µmol/L))	0.036±0.006	0.033±0.003	0.016±0.002**		0.018±0.002**
*hENT2-mediated*					
* V*_max_ (fmol/µg protein/second)	2.3±0.2	2.1±0.1	1.3±0.2**		2.0±0.2†
* K*_m_ (µmol/L)	77±23	54±12	91±33		66±25
* V*_max_ / *K*_m_ (fmol/µg protein/second/(µmol/L))	0.030±0.006	0.039±0.005	0.014±0.004**		0.030±0.007†
^hENT1/2^ *F*	1.20±0.21	0.85±0.09	1.14±0.23		0.60±0.10

hPMEC were cultured (8 hours) in presence of basal levels of insulin (*Basal-insulin*), or 1 nmol/L insulin (*Insulin*) as in Methods. Maximal velocity (*V*_max_) and apparent Michaelis-Menten constant of saturable transport (*K*_m_) were measured for hENT1- or hENT2-mediated adenosine transport. ^hENT1/2^ *F* represents the relative contribution of hENT1 and hENT2 to total adenosine transport and was estimated from *V*_max_ and apparent *K*_m_ values as described in Methods. *P<0.05 and **P<0.03 versus corresponding values in Normal. †*P*<0.05 versus corresponding values in GDM with basal-insulin.

### Adenosine transport

Total (overall) 10 µmol/L adenosine uptake (hENT1 + hENT2 mediated) was measured in absence or presence of *S*-(4-nitrobenzyl)-6-thio-inosine (NBTI, 1 µmol/L, an inhibitory concentration for hENT1 transport activity (Sigma, Atlanta, GA, USA), hypoxanthine (2 mmol/L, a nucleobase taken up via ENT2, but not via ENT1 in HUVEC and other cell types), or both, as described [1], [4]. Briefly, transport assays were performed in Krebs [(mM): NaCl 131, KCl 5.6, NaHCO_3_ 25, NaH_2_PO_4_ 1, Hepes 20, CaCl_2_ 2.5, MgCl_2_ 1 (pH 7.4, 37°C)] in cells pre-incubated (overnight) in low sera (0.2%) PCM. NBTI, hypoxanthine, unlabelled-adenosine and [^3^H] adenosine (87 nmol/L, 2,3 [^3^H] adenosine (NEN, Dreieich, FRG), 37 Ci/mmol, 2 µCi/mL, 20 seconds, 22°C) were added at *cis* compartment for influx assays as described [1]. The difference between total adenosine transport and transport in the presence of 1 µmol/L NBTI was defined as ENT1 (NBTI sensitive)-mediated adenosine transport (i.e., hereafter referred as hENT1-adenosine transport). The difference between total adenosine transport in the presence of 1 µmol/L NBTI and 2 mmol/L hypoxanthine was defined as hENT2 (NBTI insensitive)-mediated adenosine transport (i.e., hereafter referred as hENT2-adenosine transport) [3], [4].

A single Michaelis-Menten equation was used to obtain maximal velocity (*V*_max_) and apparent Michaelis-Menten constant (*K*_m_) of transport at initial rates (i.e., lineal uptake up to 20 seconds). Relative contribution of hENT1 and hENT2 (^hENT1/2^ *F*) to total transport (i.e., hENT1 + hENT2 mediated) was defined by:
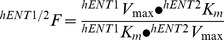
where ^hENT1^ *V*_max_ and ^hENT1^ *K*_m_ are kinetic parameters for hENT1-saturable transport, and ^hENT2^ *V*_max_ and ^hENT2^ *K*_m_ for hENT2-saturable transport. Relative effect of GDM (*GDM*) compared with normal (*N*) pregnancies on transport activity via hENT1 (1/*^N/GDM-hENT1^F*) or hENT2 (1/*^N/GDM-hENT2^F*) was estimated from maximal transport capacity (*V*_max_/*K*_m_) for transport by:



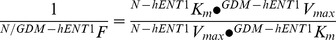
or
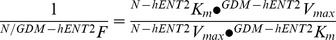
where *^N-hENT1^V*_max_, *^N-hENT2^V*_max_, *^N-hENT1^K*_m_ and *^N-hENT2^K*_m_ are kinetic parameters for transport via hENT1 and hENT2, respectively, in normal pregnancies, and *^GDM-hENT1^V*_max_, *^GDM-hENT2^V*_max_, *^GDM-hENT1^K*_m_ and *^GDM-hENT2^K*_m_ for transport via hENT1 and hENT2, respectively, in GDM. Relative contribution of insulin to saturable transport kinetic parameters was estimated from *V*_max_/*K*_m_ by:



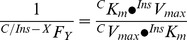
where *X* is hENT1 or hENT2-mediated transport, *Y* is normal or GDM pregnancy, *^C^V_max_* and *^C^K_m_* are transport kinetics parameters in control (basal-insulin), and *^Ins^V_max_* and *^Ins^K_m_* are in presence of insulin.

In experiments where sodium was replaced by *N*-methylglucamine-HCl (Sigma) or choline chloride (Sigma), adenosine transport was unaltered (not shown) as previously reported [1], [4]. Cell viability was assayed by Trypan blue exclusion and was not significantly altered (∼97% of viable cells) by addition of the molecules used in this study. Rinsing the monolayers with ice-cold Krebs containing 10 µmol/L NBTI and 2 mmol/L hypoxanthine terminated tracer uptake. Radioactivity in formic acid cell digests was determined by liquid scintillation counting, and uptake was corrected for [^14^C or ^3^H] mannitol (NEN) disintegrations per minute (d.p.m.) in the extracellular space [1], [4].

**Figure 3:**
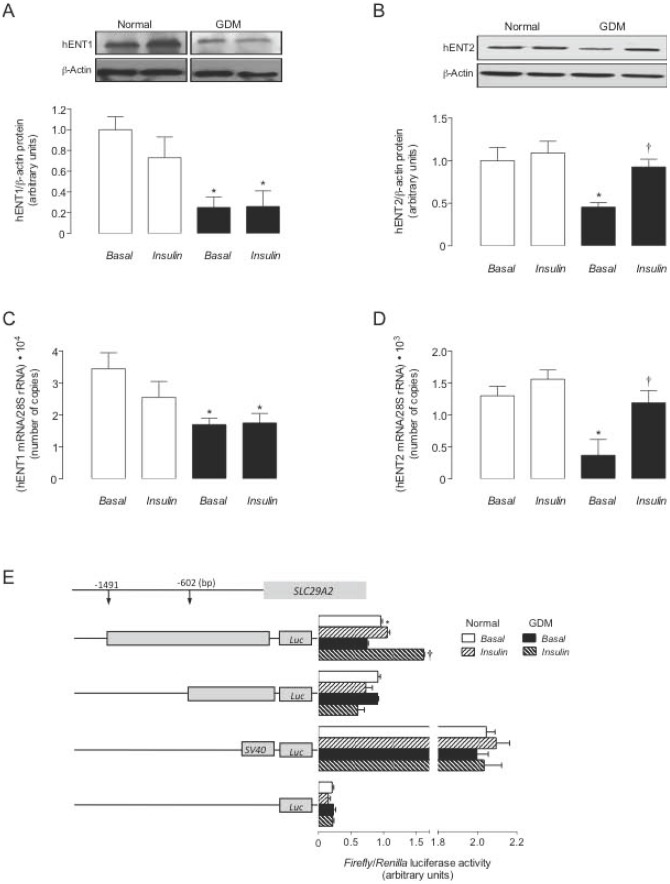
GDM and insulin effect on hENTs expression. Western blots for hENT1 (a) and hENT2 (b) in hPMEC from normal GDM pregnancies incubated (8 hours) with basal-insulin (*Basal*) or 1 nmol/L insulin (*Insulin*) (ß-actin is internal control). *Lower panel*: hENT1/ß-actin ratio densitometries from data in normal (white bars) or GDM (black bars) pregnancies, normalized to 1 in *Basal* in normal pregnancies. hENT1 (c) and hENT2 (d) mRNA expression relative to 28S rRNA as in (a) and (b). (e) Luciferase (*Luc*) reporter pGL3-hENT2^−1491^ and pGL3-hENT2^−602^ constructs activity in cells from normal or GDM pregnancies. In (a–d), *P<0.05 versus corresponding values in Normal, †*P*<0.05 versus GDM in *Basal*. In (e), *P<0.05 versus GDM for pGL3-hENT2^−1491^, †*P*<0.05 versus GDM in *Basal* for pGL3-hENT2^−1491^. Values are mean ± SEM (n = 16).

### Adenosine measurements by high-performance liquid chromatography (hplc)

Adenosine concentration was measured in whole umbilical blood (vein + arteries), umbilical arteries or veins by hplc [1], [4], [20]. For collection of vein blood, arteries from placenta-attached umbilical cord were clamped and vein blood was collected. For artery blood collection, umbilical cord was double-clamped and detached from the placenta. One end of umbilical arteries was unclamped and blood drained out. Blood samples (3.5 mL) were collected into a syringe containing (250 µL) 10 µmol/L erythro-9-(2-hydroxy-3-nonyl) adenine (EHNA, adenosine deaminase inhibitor), 10 µmol/L NBTI and 1 mmol/L dilazep (inhibitors of adenosine transport), 2 µg/mL indomethacin (inhibitor of nucleotides release from platelets), and 40 µmol/L *O*,*O*'-bis(2-aminoethyl)ethyleneglycol-*N*,*N*,*N*'-*N*'-tetraacetic acid (G-EDTA, inhibitor of adenosine release from platelets) immediately after birth, as described [4], [19]. Samples were centrifuged (14,000 *g*, 1 minute) and aliquots (1 mL) of plasma were deproteinated (100 µL, 50% trichloroacetic acid) and centrifuged again (5 minutes). Supernantant (750 µL) was neutralized by addition of 100 µL of 3.3 N potassium hydroxide. Adenine nucleotides were extracted by adding 500 µL of 1 mol/l zinc sulfate and 1 mL of saturated barium hydroxide, and vortex mixed for 10 seconds, centrifugated (14,000 *g*, 5 minutes). Adenosine was finally converted to ethenoadenosine by mixing the sample with chloroacetaldehyde to a final concentration of 440 mmol/L and incubating it at 80°C for 1 hour. Derivatized samples were mixed and stored at 4°C until use 60–120 minutes later for hplc analysis.

**Figure 4:**
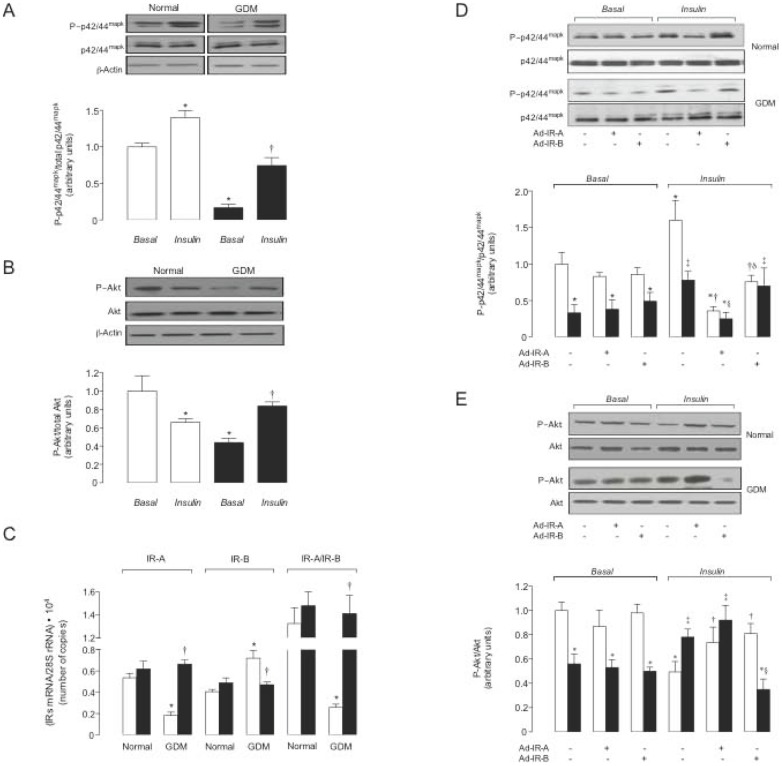
GDM and insulin effect, and involvement of IR isoforms on p42/44^mapk^ and Akt phosphorylation. Western blot for phosphorylated (P∼p42/44^mapk^) or total (p42/44^mapk^) p42/44^mapk^ (a) and phosphorylated (P∼Akt) or total (Akt) Akt (b) in hPMEC from normal or GDM pregnancies incubated (8 hours) with basal-insulin (*Basal*) or 1 nmol/L insulin (*Insulin*) (ß-actin is internal control). *Lower panels*: P∼p42/44^mapk^/total p42/44^mapk^ and P∼Akt/total Akt ratio densitometries from data in normal (white bars) or GDM (black bars) pregnancies, normalized to 1 in *Basal* in normal pregnancies. (c) IR-A and IR-B mRNA expression relative to 28S rRNA (internal reference) in the presence of basal-insulin (white bars) or 1 nmol/L insulin (black bars). (d, e) Western blots in not-transduced (–) or transduced (+) cells with adenovirus siRNA against insulin receptor isoform A (Ad-IR-A) or B (Ad-IR-B) in cells from normal (white bars) or GDM (black bars) pregnancies as in (a). In (a, b) (n = 16), *P<0.05 versus Normal in *Basal*, †*P*<0.05 versus GDM in *Basal*. In (c) (n = 16), *P<0.05 versus corresponding values in Normal in *Basal*, †*P*<0.05 versus GDM in *Basal*. In (d) (n = 10) for *Basal*, *P<0.05 versus corresponding values in Normal; for *Insulin*, *P<0.05 versus Normal not-transduced cells in *Basal*, †*P*<0.05 versus Normal not-transduced cells, ‡*P*<0.05 versus GDM not-transduced cells in *Basal*, §*P*<0.05 versus GDM not-transduced or Ad-IR-B transduced cells, δ*P*<0.05 versus Normal Ad-IR-A transduced cells. In (e) (n = 10) for *Basal*, *P<0.05 versus corresponding values in Normal; for *Insulin*, *P<0.05 versus Normal not-transduced or transduced cells in *Basal*, †*P*<0.05 versus Normal not-transduced cells, ‡*P*<0.05 versus GDM not-transduced or transduced cells in *Basal*, §*P*<0.05 versus GDM not-transduced or Ad-IR-A transduced cells. Values are mean ± SEM.

For hplc analysis of samples, aliquots (200 µL) were collected and mixed with 10 µL of 0.5 mol/L acetate-buffer, 10 µL of 1 µmol/L internal standard (adenosine), and 10 µmol/L of 50% aqueous chloroacetaldehyde. After incubation (80°C, 1 hour) and centrifugation (14,000 *g*, 4 minutes), aliquots (80 µL) were injected into an Isco hplc system (pump model 2350, gradient programmer model 2360, 4.6×250 mm C_18_ reverse-phase column, 5-µm particle size) (Chemical Research Data Management System, Lincoln, NE, USA). Mobile phase was 10 mmol/L citrate-buffer with 4.5% acetonitrile and was run isocratically at 1 mL/minute. Fluorescence detection was achieved at an excitation wavelength of 275 nm and an emission wavelength of 420 nm using a Waters M-470 fluorescence detector. Ratio of the area under the adenosine peaks to the area under the internal standard peak was compared with a standard curve [1], [4]. The concentration of adenosine was calculated from the peak area, using the standard line. Pearson's correlation coefficient for the standard line of standard adenosine solution was more than 0.999 from 2.997 to 431 nmol/L, and the recovery of plasma adenosine was 82.7±1.0% (n = 4−5). Plasma adenosine concentrations were calculated by dividing the amount of adenosine in the samples by the volume of plasma assayed, as described [4].

**Figure 5:**
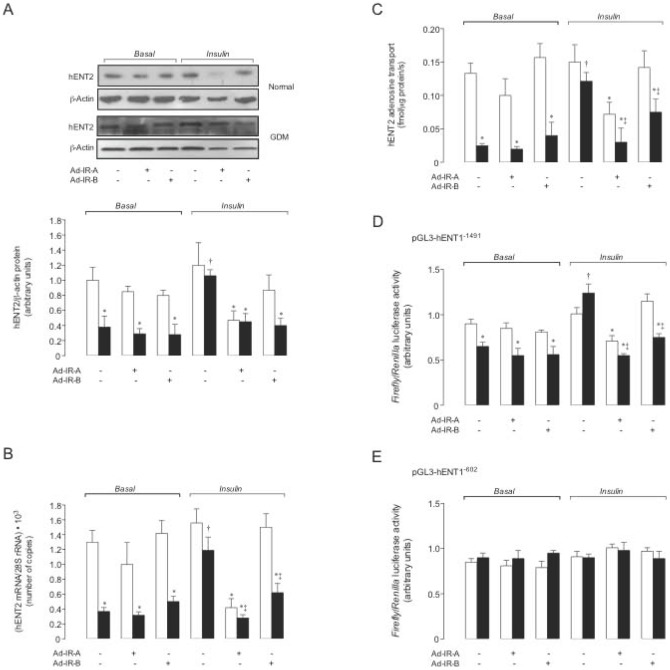
Insulin blocks GDM-reduced hENT2-mediated adenosine transport requiring IR-A and IR-B expression. Western blot (a) and mRNA expression (b) for hENT2 in hPMEC from normal or GDM pregnancies incubated (8 hours) with basal-insulin (*Basal*) or 1 nmol/L insulin (*Insulin*), in not-transduced (–) or transduced (+) cells with adenovirus siRNA against insulin receptor isoform A (Ad-IR-A) or B (Ad-IR-B) (ß-Actin and 28S rRNA were internal controls). *Lower panel*: hENT2/ß-actin ratio densitometries derived from data in normal (white bars) or GDM (black bars) pregnancies, normalized to 1 in not-transduced cells from normal pregnancies in *Basal*. (c) hENT2-mediated adenosine (10 µmol/L) transport as in (a). Luciferase (*Luc*) reporter pGL3-hENT2^−1491^ (d) and pGL3-hENT2^−602^ (e) constructs activity. In (a–d) for *Basal*, *P<0.05 versus corresponding values in Normal; for *Insulin*, *P<0.05 versus Normal or GDM not-transduced or Normal IR-B transduced cells, †*P*<0.05 versus all values in GDM. In (b), (c), and (d) for *Insulin*, ‡*P*<0.05 versus GDM IR-B transduced cells in *Insulin*. Values are mean ± SEM (n = 16).

### Reverse transcription and quantitative RT-PCR

Total RNA was isolated using the Quiagen RNAeasy kit (Quiagen, Crawley, UK). RNA quality and integrity were insured by gel visualization and spectrophotometric analysis (OD_260/280_), quantified at 260 nm and precipitated to obtain 4 µg/µL. Aliquots (1 µg) of total RNA were reversed transcribed into cDNA as described [4], [21].

Quantitative RT-PCR (qRT-PCR) was performed using a LightCycler^TM^ rapid thermal cycler (Roche Diagnostics, Lewes, UK) in a reaction mix containing 0.5 µmol/L primers, and dNTPs, *Taq* DNA polymerase and reaction buffer provided in the QuantiTect SYBR Green PCR Master Mix (QUIAGEN, Crawley, UK) [1], [4], [21]. HotStart *Taq* DNA polymerase was activated (15 minutes, 95°C), and assays included a 95°C denaturation (15 seconds), annealing (20 seconds) at 58°C (hENT1), 57°C (hENT2), 60°C (IR-A), 60°C (IR-B), 56°C (28 S), and extension at 72°C (hENT1 15 seconds, hENT2 20 seconds, IR-A 20 seconds, IR-B 20 seconds, 28S 10 seconds). Fluorescent product was detected after 3-seconds step to 5°C below the product melting temperature (*T*_m_). Product specificity was confirmed by agarose gel electrophoresis (2% w/v) and melting curve analysis. The product *T*_m_ values were 79.5°C for hENT1, 85.5°C for hENT2, 87.2°C for IR-A, 87.6°C for IR-B and 82.4°C for 28S. hENT1, hENT2, IR-A, IR-B and 28S standards were prepared as described [1], [4]. Oligonucleotide primers: hENT1 (sense) 5′-TCTCCAACTCTCAGCCCACCAA-3′, hENT1 (*anti*-sense) 5′-CCTGCGATGCTGGACTTGACCT-3′, hENT2 (sense) 5′-TCTCCAACTCTCAGCCCACCAA-3′, hENT2 (*anti*-sense) 5′-CCTGCGATGCTGGACTTGACCT-3′, IR-A (sense) 5′-GCTGAAGCTGCCCTCGAGGA-3′, IR-A (*anti*-sense) 5′-CGAGATGGCCTGGGGACGAA-3′, IR-B (sense) 5′-GCTGAAGCTGCCCTCGAGGA-3′, IR-B (*anti*-sense) 5′-AGATGGCCTAGGGTCCTCGG-3′, 28S (sense) 5′-TTGAAAATCCGGGGGAGAG-3′, 28S (*anti*-sense) 5′-ACATTGTTCCAACATGCCAG-3′. Expected size products for hENT1 (151 bp), hENT2 (209 bp) IR-A (210 bp), IR-B (244 bp) and 28S (100 bp) were confirmed in PCR experiments. The 28S rRNA number of copies was unaltered (*P*>0.05, n = 6) in all experimental conditions (not shown).

**Table 3:** Effect of GDM and insulin on hENT1 and hENT2 relative contribution to total adenosine transport.

Parameter	
1/*^N/GDM-hENT1^F*	0.44±0.06
1/*^N/GDM-hENT2^F*	0.47±0.11
1/*^C/Ins-hENT1^F_N_*	0.92±0.10
1/*^C/Ins-hENT2^F_N_*	1.30±0.20
1/*^C/Ins-hENT1^F_GDM_*	1.13±0.14
1/*^C/Ins-hENT2^F_GDM_*	2.15±0.50

hPMEC from normal (*N*) or gestational diabetes (*GDM*) pregnancies were cultured (8 hours) in the presence of basal levels of insulin (*C*) or 1 nmol/L insulin (*Ins*) and kinetic parameters for hENT1- or hENT2-mediated adenosine transport (i.e., maximal velocity (*V*_max_) and apparent Michaelis-Menten constant of saturable transport (*K*_m_)) measured as described in Methods. The *V*_max_ and apparent *K*_m_ values were used for estimation of *F* values (see Methods).

### Western blotting

Proteins (70 µg) separated by polyacrylamide gel (10%) electrophoresis were probed with primary polyclonal goat *anti*-hENT1 (1∶1000) or *anti*-hENT2 (1∶1000) (Santa Cruz Biotechnology, USA), rabbit *anti*-p42/44^mapk^ (1∶1500), mouse *anti*-phosphorylated p42/44^mapk^ (P∼p42/44^mapk^, 1∶000), rabbit *anti*-Akt (1∶1500), rabbit *anti*-phosphorylated Akt (P∼Akt, 1∶250) (Cell Signaling, USA), or monoclonal mouse *anti*-insulin receptor ß-subunit (IRß, 1∶1500) (Santa Cruz Biotechnology) and *anti*-ß-actin (1∶2000) (Santa Cruz Biotechnology) antibodies followed by incubation (1 hour) in Tris buffer saline Tween/0.2% bovine serum albumin containing secondary horseradish peroxidase-conjugated goat *anti*-goat, -rabbit or -mouse antibodies (Santa Cruz Biotechnology) as described [1], [4], [21]. Proteins were detected by enhanced chemiluminescence (film exposure time was 5 minutes) and quantitated by densitometry.

**Figure 6:**
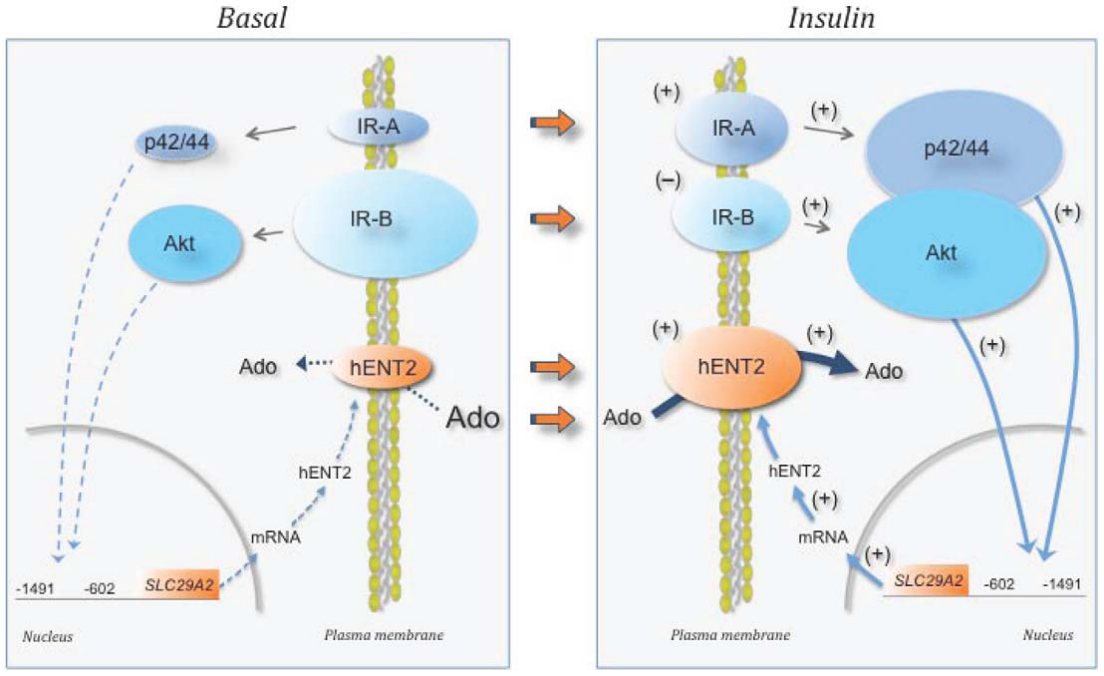
Proposed action model of insulin on hPMEC from GDM. hPMEC from GDM pregnancies in presence of basal-insulin (*Basal*) exhibit reduced expression of insulin receptor A (IR-A), but increased expression of insulin receptor B (IR-B). In addition, hENT2 protein abundance is reduced leading to lower removal (dotted arrow) of extracellular adenosine (Ado), thus leading to extracellular accumulation of adenosine. Altered IR-A and IR-B expression associates with preferential reduction of p42/44^mapk^ (p42/44) activation compared with Akt activation, respectively, favouring a metabolic- rather than a mitogenic-like phenotype. This phenomenon leads to reduced signalling (segmented light blue arrows) mediated by p42/44 and Akt, respectively, reducing promoter activity between −1491 and −602 bp from ATG of *SLC29A2* (for hENT2). Thus, lowered mRNA expression and hENT1 protein abundance could explain a reduced hENT2 availability at the plasma membrane to take up adenosine. When hPMEC cultures are exposed to insulin (*Insulin*), GDM-reduced IR-A and increased IR-B is reversed (orange arrows) to similar expression levels (+, increase; –, decrease), reaching comparable values to normal pregnancies under basal-insulin. Furthermore, IR-A and IR-B altered expression associates with restoration of p42/44^mapk^ and Akt activation, respectively, resulting in p42/44^mapk^/Akt ∼1 (i.e., similar contribution of p42/44^mapk^ and Akt signalling pathways), thus recovering the equilibrium between mitogenic and metabolic phenotype characteristic of hPMEC from normal pregnancies. These changes lead to restored signalling (solid light blue arrows) mediated by p42/44 and Akt, respectively, causing activation of *SLC29A2* promoter activity and normal hENT2 mRNA expression and protein synthesis increasing this transporter availability at the plasma membrane. The latter restores hENT2-mediated transport of adenosine from the extracellular space reestablishing extracellular levels of this nucleoside.

### hENT2 mRNA and protein levels half-life

Total RNA and protein from hPMEC exposed (0–24 hours) to culture medium without or with 1.5 μmol/L actinomycin D (transcription inhibitor) or 1 μmol/L cycloheximide (protein synthesis inhibitor) was measured as described [21]. hENT2 and 28S mRNA were amplified by qRT-PCR, and hENT2 and ß-actin proteins detected by western blot.

### hENT2 promoter cloning

Genomic DNA was isolated using the Wizard® SV Genomic DNA Purification System (Promega, Madison, WI, USA). Upstream sequences to ATG translation start codon of *SLC29A2* gene (GeneBank accession: AF034102) were amplified by PCR using Elongase® Enzyme System (Invitrogen, USA) [21] following manufacturer instructions. The oligonucleotide primers were: hENT2S1 (sense) 5′-ACGCGGTTAGCAACCTGTTAACA-3′, hENT2S2 (sense) 5′-ACGCGTTACGCTCTGGGCGGGGAC-3′, hENT2S3 (*anti*-sense) 5′-CCATGGCGCGAGGAGACGCC'. Amplification products of 1491 and 602 bp were purified from an agarose gel using QIAquick Gel Extraction kit (Qiagen, USA) [4], [21]. Products were then cloned into pCR®-Blunt II-TOPO® (Invitrogen, USA) and fully sequenced using BigDye Terminator v3.1 Cycle Sequencing kit (Applied Biosystems) and the automated ABI Prism 3730 DNA sequencer (Applied Biosystems/Hitachi). Constructs were digested with *Mlu*I and *Nco*I restriction enzymes and inserts were detected in an agarose gel and purified as above. The resulting sticky-end inserts were subcloned into pGL3-Basic vector (Promega, USA) by ligation (4°C, 16 hours) using T4 DNA Ligase (Promega, USA) in *Mlu*I and *Nco*I restriction sites upstream *Firefly* luciferase reporter gene. Generated reported constructs were pGL3-hENT2^−1491^ and pGL3-hENT2^−602^.

### Transient transfection

Sub-confluent (75%) hPMEC primary cultures were resuspended in serum-free M199. Cell suspension (3.2×10^6^ cells/mL) was mixed with 10 µg of pGL3-hENT2^−1491^ or pGL3-hENT2^−602^ reporter constructs, pGL3-Basic (empty pGL3 vector), pGL3-Control (Simian Virus 40 promoter (SV40) pGL3 vector) or the internal transfection control vector pRL-TK expressing *Renilla* luciferase (Promega), as described [4], [21]. Cells were then electroporated (300 Volts, 700 µF, 5–10 milliseconds)(Gene Pulser® II System, BioRad, CA, USA) and cultured in M199 containing 2% FCS for 48 hours before experiments. Transfection efficiency was estimated by transfection of the pEGFP-N3 vector (Clontech, Mountain View, CA, USA) and fluorescent cells were counted under a fluorescent-equipped inverted microscope (Leica DMIL; Wetzlar, Germany) as described [4], [21].

### Luciferase assay

Electroporated cells were lysed in 200 µL passive lysis buffer (Promega), and *Firefly* and *Renilla* luciferase activity was measured using Dual-Luciferase® Reporter Assay System (Promega) in a Sirius luminometer (Berthold Detection System; Oak Ridge, TN, USA) as described [4], [21].

### Insulin receptor isoforms suppression with small interference RNA

To suppress IR-A and IR-B expression an adenoviral-based siRNA delivering system (pSilencer™ adeno 1.0-CMV System Kit, Ambion) was used. Complementary oligonucleotides for IR-A (sense: 5′-GTTTTCGTCCCCAGGCCATCTTTCAAGAGAAGATGGCCTGGGGACGAAAAC-3′, *anti*-sense: 5′- CAAAAGCAGGGGTCCGGTAGAAAGTTCTCTTCTACCGGACCCCTGCTTTTG-3′) and IR-B (sense: 5′- GACCCTAGGCCATCTCGGAAATTCAAGAGATTTCCGAGATGGCCTAGGGTC-3, anti-sense: CTGGGATCCGGTAGAGCCTTTAAGTTCTCTAAAGGCTCTACCGGATCCCAG-3′) encoding for a siRNA hairpin targeting human IR (GenBank accession: NM_001079817.1 for IR-A and NM_000208.2 for IR-B) were designed, annealed and cloned into a pShuttle vector. Ligation products were amplified in *E. Coli* DH5α competent cells by bacterial transformation and plasmid constructs were confirmed by restriction enzyme digestion. The pShuttle-IRA/B siRNA and the adenoviral LacZ backbone were linearized following PacI digestion and were transfected into HEK-293 cells to generate the IR siRNA recombinant adenovirus(Ad-siIRA and Ad-siIRB). The negative control pShuttle vector (encoding a scramble siRNA sequence not found in human, mouse or rat genome databases) was used to generate the negative control adenovirus. A positive siGAPDH provided by the kit was also used to generate an Ad-siGAPDH. Recombinant adenoviral vectors were expanded by serial infection of HEK-293 cells, harvested by a three freeze-thaw procedure and used to infect primary cultures (passage 1) of hPMEC. Adenoviral particles were purified and quantified before experiments using a commercial kit (ViralBind™ Adenovirus Purification kit, Cell Biolabs, USA). Cells at 50–60% confluence were seeded 24 hours before adenovirus infection. Viral stocks were diluted to reach the desired multiplicity of infection (MOI) in serum-free medium and added to the cell monolayer. Infected cells were incubated with serum free medium for 8 hours. After this period the infective medium was changed to complete culture medium and cells were incubated for a further 48 hours under standard culture conditions. Isolation of total RNA and protein, and functional assays were then performed as above.

### Statistical analysis

Values are mean ± SEM, with different cell cultures (2–4 replicates) from normal (n = 64) or GDM (n = 64) pregnancies. Since the yield of hPMEC from one single placenta was not enough to proceed with all the experimental strategies included in this study, the reported n values is variable and corresponds to paired cell cultures from normal and GDM pregnancies. Data reported in this study describe a normal standard distribution and comparison between two and more groups were performed by means of Student's unpaired *t*-test and analysis of variance (ANOVA), respectively. If the ANOVA demonstrated a significant interaction between variables, *post hoc* analyses were performed by the multiple-comparison Bonferroni correction test. The statistical software GraphPad Instat 3.0b and Graphpad Prism 5.0b (GraphPad Software Inc., San Diego, CA, USA) were used for data analysis. *P*<0.05 was considered statistically significant.

## Results

### Patients and newborns

Patients with GDM exhibit increased maternal glycosilated hemoglobin A_1c_, altered OGTT, insulinemia, increased insulin resistance [21] and reduced ß-cell function (Table 1). In addition, increased fetal insulin resistance and ponderal index in GDM compared with normal pregnancies was found.

### Adenosine transport

Overall adenosine transport was lower in GDM compared with normal pregnancies (Fig. 1a). NBTI reduced transport in cells from normal pregnancies to values in GDM, and hypoxanthine reduced adenosine transport only in normal pregnancies. NBTI reduced adenosine transport in GDM to values in normal or GDM pregnancies in presence of NBTI + hypoxanthine. Fig. 1a also shows that insulin did not alter overall adenosine transport in normal pregnancies, but reduced GDM-inhibition of transport. NBTI and hypoxanthine reduced adenosine transport only in normal pregnancies, an effect that was comparable to GDM-inhibition in presence of insulin. However, hypoxanthine reduced transport in GDM to values in normal or GDM pregnancies with NBTI + hypoxanthine.

Knowing that hENT1 and hENT2 are differentially regulated by insulin in placental macrovascular endothelium from GDM [5], [9], we assayed whether GDM and insulin effect regards selective hENTs modulation in hPMEC. GDM reduced hENT1- and hENT2-adenosine transport compared with normal pregnancies (Fig. 1b). Insulin did not alter hENT1- or hENT2-adenosine transport in normal pregnancies or the GDM-inhibited hENT1 transport; however, insulin reversed GDM-inhibited hENT2-adenosine transport to values in normal pregnancies.

### Umbilical blood adenosine concentration

Adenosine concentration in umbilical whole (arteries + veins) and vein blood was higher in GDM (Fig. 1c). However, adenosine concentration in umbilical arteries blood was similar in GDM compared with normal pregnancies.

### Adenosine transport kinetic parameters

hENT1- and hENT2-mediated transport was saturable (Fig. 2a, c) and lineal in a Eadie-Hofstee analysis (Fig. 2b, c). The *V*_max_ for hENT1- or hENT2-mediated transport was lower in GDM compared with normal pregnancies, without significant changes in apparent *K*_m_ (Table 2). Insulin blocked GDM effect on hENT2, but did not alter kinetic parameters for hENT1-adenosine transport in GDM or normal pregnancies.

### hENT1 and hENT2 expression

hENT1 and hENT2 protein abundance was lower in GDM versus normal pregnancies (Fig. 3a). Insulin blocked GDM effect on hENT2 protein abundance (Fig. 3b) or mRNA number of copies (Fig. 3d). However, insulin did not alter hENT1 expression in both cell types (Fig. 3a, c). hENT2 protein and mRNA number of copies half-life were unaltered in cells from GDM in absence or presence of insulin compared with cells from normal pregnancies (not shown).

### *SLC29A2* promoter activity

Reporter luciferase activity in cells from GDM transfected with pGL3-hENT2^−1491^, but not pGL3-hENT2^−602^ construct was lower compared with normal pregnancies in basal-insulin (Fig. 3e). Insulin did not alter reporter activity in pGL3-hENT2^−1491^-transfected cells from normal pregnancies, but reversed GDM-reduced pGL3-hENT2^−1491^ reporter activity to values determined in cells from normal pregnancies in basal-insulin.

### Insulin signalling

Since signalling pathways activated by insulin involve p42/44^mapk^ and Akt activation, phosphorylation of these molecules was assayed. Ratios for P∼p42/44^mapk^/p42/44^mapk^ (Fig. 4a) and P∼Akt/Akt (Fig. 4b) in presence of basal levels of insulin were lower in GDM compared with normal pregnancies, an effect less pronounced for P∼Akt/Akt compared with P∼p42/44^mapk^/p42/44^mapk^. Insulin increased P∼p42/44^mapk^/p42/44^mapk^, but reduced P∼Akt/Akt ratios in normal pregnancies; however, blocked GDM effect on these molecules.

### IR isoforms expression

IR-A and IR-B mRNA expression was comparable in both cell types (Fig. 4c). IR-A mRNA expression was reduced, but IR-B mRNA expression was increased, leading to lower IR-A/IR-B ratio, in GDM compared with normal pregnancies. GDM effect was blocked by insulin, but IR-A/IR-B mRNA expression was unaltered by this hormone in normal pregnancies.

Neither IR-A nor IR-B knockdown altered P∼p42/44^mapk^/p42/44^mapk^ (Fig. 4d) or P∼Akt/Akt (Fig. 4e) ratios under basal-insulin in both cell types. However, insulin-increased P∼p42/44^mapk^/p42/44^mapk^ and reduced P∼Akt/Akt was blocked in IR-A or IR-B knockdown cells from normal pregnancies, respectively. Insulin restoration of GDM-reduced P∼p42/44^mapk^/p42/44^mapk^ was absent in IR-A knockdown cells (Fig. 4d). However, IR-B knockdown blocked insulin restoration of GDM-reduced P∼Akt/Akt (Fig. 4e). IR-A or IR-B supression did not alter hENT2 expression (Fig. 5a, b) and activity (Fig. 5c), and *SLC29A1* promoter constructs activity (Fig. 5d, e) in cells from normal or GDM pregnancies under basal-insulin. IR-A or IR-B supression blocked insulin effect on hENT2 expression and activity, and pGL3-hENT1^−1491^ construct promoter activity; however, insulin did not alter pGL3-hENT1^−602^ in GDM.

## Discussion

This study shows for the first time that GDM is a syndrome associated with reduced overall adenosine transport due to reduced hENT1 and hENT2 activity in primary cultures of human placental microvascular endothelial cells (hPMEC). Since there is increasing experimental evidence establishing that macrovascular compared with microvascular endothelial cell function is different within a same vascular bed (in this case, the human placental vasculature), a characteristic that is critical for the vascular hemostasis, we believe that our present study contributes to a better understanding of the differential biological functions of hPMEC in health and disease. The results of this study also show increased umbilical vein, but not arteries blood adenosine concentration compared with normal pregnancies. Insulin reestablishes GDM-reduced hENT2, but not hENT1 expression and activity. hPMEC from GDM exhibit lower IR-A/IR-B mRNA expression and p42/44^mapk^/Akt activity ratios compared with normal pregnancies, an effect also blocked by insulin. IR-A or IR-B knockdown blocked insulin-restored GDM-reduced hENT2 protein abundance, mRNA expression and activity; however, only IR-A knockdown reduced hENT2 expression and activity in normal pregnancies. Regarding hENTs-mediated adenosine transport, insulin reverses a GDM- (preferentially metabolic) to a normal (preferentially mitogenic)-phenotype via IR-B and IR-A differential expression and activation in hPMEC.

### Adenosine transport

Overall adenosine transport was reduced in hPMEC from GDM compared with normal pregnancies, an effect likely due to reduced hENT1- and hENT2-mediated transport. These results complement the elevated umbilical vein blood adenosine concentration recently reported by our group in umbilical vein blood in GDM [4]. We here show that plasma adenosine concentration in umbilical arteries was unaltered in GDM, suggesting that increased adenosine detected in umbilical vein blood may result from reduced adenosine uptake in hPMEC. In addition, since umbilical artery carries blood from the fetus to the placenta, the latter findings could be the result of a defective placental rather than fetal vasculature adenosine handling in GDM. However, a higher placental adenosine release and/or elevated fetal extracellular adenosine catabolism in GDM can not be rulled out.

Kinetics of adenosine transport show that relative basal maximal transport capacity (*V*_max_/*K*_m_) values for hENT1 and hENT2 are similar in normal or GDM (^hENT1/2^ *F* ∼1.1) pregnancies (see Table 2). Therefore, the reported comparable contribution to overall adenosine transport of these proteins in hPMEC from normal pregnancies [1] is apparently unaltered by GDM. The *V*_max_/*K*_m_ values for hENT1 and hENT2 are similarly reduced (∼55%) (see 1/*^N/GDM-hENT1^F* and 1/*^N/GDM-hENT2^F* values in Table 3) due to lower *V*_max_ in GDM compared with normal pregnancies. Since this GDM effect was associated with similar reduction (∼65%) in hENT1 and hENT2 protein abundance, with no changes in the half-life of these proteins, GDM-reduced transport may result from hENT1 and hENT2-lower availability rather than reduced affinity of a fix number of transporters at the plasma membrane [22].

GDM is also associated with lower (∼62%) hENT1 and hENT2 mRNA expression, and unaltered mRNA half-lifes, suggesting that reduced availability of these membrane transporters may results from reduced *SLC29A1* (for hENT1) and *SLC29A2* (for hENT2) transcription. Supporting the latter a correlation between GDM-reduced adenosine transport and transporters expression (transport activity/protein abundance, transport activity/mRNA expression, and protein abundance/mRNA expression ratios were ∼1.04) was found. Results on *SLC29A2* transcriptional activity suggest that a repressor consensus sequence is feasible to exist within −1491 and −602 bp from ATG of this gene in hPMEC from GDM pregnancies. On the contrary, the −602 bp promoter fragment is apparently not involved in the GDM-reduced expression of *SLC29A2* in this cell type.

### Insulin effect on adenosine transport

GDM-reduced hENT2-, but not hENT1-mediated adenosine transport was reversed by insulin, suggesting differential modulation of these membrane transporters. Since insulin action was measured in hPMEC cultured in presence of insulin concentrations equivalent to those found in whole umbilical blood from GDM or normal pregnancies at birth, the possibility that insulin effect was due to this hormone withdrawal in culture is unlikely. Restoration of GDM-reduced hENT2 activity by insulin resulted from a higher *V*_max_/*K*_m_ (∼1.7 fold, from (1/*^C/Ins-hENT2^F_GDM_*)/(1/*^C/Ins-hENT2^F_N_*) in Table 3). Most likely insulin effect on hENT2 transport results from restablishment of hENT2 expression since insulin increased the abundance of this protein (∼2 fold) and mRNA expression (∼3.5 fold). The finding that insulin effect on hENT2 mRNA expression is higher (∼1.7 fold) than the protein abundance, suggests that hPMEC from GDM could require higher *SLC29A2* transcriptional activity to sustain hENT2 protein content. In addition, because insulin restores GDM-reduced *SLC29A2* transcriptional activity and modulates different transcription factors [23], this hormone could also be acting as potential inhibitor of repressive transcription factor(s) in *SLC29A2* in GDM. Interestingly, insulin was innefective in reversing GDM effects on hENT1 expression and activity in hPMEC. This finding suggests that this nucleoside transporter isoform is likely not under regulation by insulin in this cell type. This is a result complemented by findings in rat B lymphocytes where rat ENT1 (rENT1) is unaltered, but rENT2 is increased by insulin [24]. Thus, a differential modulation of hENT1 and hENT2 by insulin is not exclusive of human placental endothelial cells. Furthermore, insulin was also innefective in modulating the expression or activity of hENT1 in cells from normal pregnancies. The latter could be a characteristic of hPMEC that may be associated with its microvascular origin compared with cells derived from the macrovasculature of the human placenta.

### IR isoforms role

IR-A (associated with a mitogenic phenotype) and IR-B (associated with a metabolic phenotype) [15] are expressed in human placenta [5], [9], including HUVEC [4]. We now show that IR-A and IR-B mRNA are also expressed in hPMEC primary cultures, where IR-A is the predominant isoform (IR-A/IR-B mRNA ∼5.3) resulting from reduced IR-A mRNA expression in normal pregnancies, but increased IR-B mRNA expression in GDM. Thus, hPMEC from GDM exhibit a predominant metabolic-like phenotype, which is supported by a higher reduction in p42/44^mapk^ activation, but reduced Akt activation in this cell type. Interestingly, these findings contrast with our own findings of unaltered IR-B, but increased IR-A mRNA expression in HUVEC from GDM [4]. Thus, GDM may triggers IR isoforms differential expression depending on whether the endothelium is from placental macro or microvasculature [5], [9].

Since preferential IR-B over IR-A expression seems associated with insulin resistance in patients with T2DM [12], [13], hPMEC phenotype from GDM could suggest insulin resistance [4], [5], [7]. Supporting the latter are reports showing that HUVEC from GDM required more insulin (∼2.1 fold) compared with cells from normal pregnancies to alter adenosine transport, and increased fetal insulin resistance and subsequent reduced maternal and fetal insulin sensitivity in GDM [25]. Interestingly, even when the fetuses from GDM courses with supraphysiological hyperinsulinemia (∼0.07 nmol/L) the results showing reduced IR-A, but increased IR-B mRNA expression in the presence of basal insulinemia could result from a stage of insulin resistance of hPMEC (HOMA-IR GDM/HOMA-IR normal ∼2.4, see Table 1). The biological actions of a concentration of insulin higher (1 nmol/L) than the insulinemia for GDM result in restoration of this syndrome-associated alterations of IR-A and IR-B mRNA expression to values in cells from normal pregnancies. This finding suggests that basal insulin concentration may not be enough to cause a significant change in IR-A and IR-B mRNA expression in hPMEC from GDM. However, cells from GDM will need higher than basal concentration of this hormone to get its physiological effect. In addition, a higher p42/44^mapk^/Akt ratio (higher mitogenic/metabolic-like signalling ratio) is characteristic of a higher IR-A/IR-B ratio, confirming differential cell signalling activation in response to insulin [15], [26]. Since insulin blocked GDM effect on IR isoforms expression and P∼p42/44^mapk^/p42/44^mapk^ and P∼Akt/Akt ratios, we suggest that hPMEC from GDM are likely to be resistant to insulin.

Insulin could act reversing a metabolic- to a mitogenic-like predominant phenotype. Complementing the latter, P∼p42/44^mapk^/p42/44^mapk^ and P∼Akt/Akt ratios, and hENT2 mRNA expression, protein abundance and transport activity were unaltered in IR-A or IR-B knockdown cells from normal or GDM pregnancies in basal levels of insulin. Thus, basal expression of IR isoforms itself may not be involved in modulating hENT2-adenosine transport in hPMEC. On the contrary, since insulin restoration of GDM-reduced P∼p42/44^mapk^/p42/44^mapk^ and P∼Akt/Akt ratios was abolished by supression of IR-A and IR-B expression, respectively, IR subtype-specific response and differential role in hPMEC from GDM is suggested. Interestingly, insulin activation of IR-A leads to preferential activation of a metabolic- rather than a mitogenic-like signalling pathway in response to IR-B activation in the mouse embrionic fibroblast cell line R^–^ [11]. However, an apparent common role for IR-A or IR-B isoforms expression is likely in hPMEC from GDM since supression of any of these isoforms blocked insulin restoration of GDM-reduced hENT2 transport activity and expression.

In summary, we here report the first evidences showing that hPMEC from GDM pregnancies exhibit abnormal adenosine uptake, which could result from reduced hENT1- and hENT2-transport activity and expression. This could explain the elevated umbilical vein blood adenosine concentration detected in GDM. It is proposed that supraphysiological levels of insulin are required to reverse a ‘GDM-like phenotype’ to a ‘normal-like phenotype’ in hPMEC (Fig. 6). In addition, cells from GDM exhibit higher IR-A/IR-B ratio due to reduced IR-A, but increased IR-B mRNA expression, in parallel with reduced p42/44^mapk^/Akt activity ratio, suggesting a metabolic- rather than a mitogenic-like phenotype in this cell type. Insulin restoration of these alterations results in normalization of adenosine transport due to restablishment of both IR isoforms expression to levels in cells from normal pregnancies. Since GDM is associated with maternal and fetal insulin resistance [5], [7], a crucial role of IR isoforms is proposed modulating hPMEC biological function in the human placenta circulation.
